# Plastome Evolution in *Dolomiaea* (Asteraceae, Cardueae) Using Phylogenomic and Comparative Analyses

**DOI:** 10.3389/fpls.2020.00376

**Published:** 2020-04-15

**Authors:** Jun Shen, Xu Zhang, Jacob B. Landis, Huajie Zhang, Tao Deng, Hang Sun, Hengchang Wang

**Affiliations:** ^1^CAS Key Laboratory of Plant Germplasm Enhancement and Specialty Agriculture, Wuhan Botanical Garden, Chinese Academy of Sciences, Wuhan, China; ^2^Center of Conservation Biology, Core Botanical Gardens, Chinese Academy of Sciences, Wuhan, China; ^3^University of Chinese Academy of Sciences, Beijing, China; ^4^Department of Botany and Plant Sciences, University of California, Riverside, Riverside, CA, United States; ^5^School of Integrative Plant Science, Section of Plant Biology and the L.H. Bailey Hortorium, Cornell University, Ithaca, NY, United States; ^6^Key Laboratory for Plant Diversity and Biogeography of East Asia, Kunming Institute of Botany, Chinese Academy of Sciences, Kunming, China

**Keywords:** *Dolomiaea*, plastomes, alpine environments, phylogenomic framework, highly divergent regions, positive selection, adaptive evolution

## Abstract

*Dolomiaea* is a medicinally important genus of Asteraceae endemic to alpine habitats of the Qinghai-Tibet Plateau (QTP) and adjacent areas. Despite significant medicinal value, genomic resources of *Dolomiaea* are still lacking, impeding our understanding of its evolutionary history. Here, we sequenced and annotated plastomes of four *Dolomiaea* species. All analyzed plastomes share the gene content and structure of most Asteraceae plastomes, indicating the conservation of plastome evolutionary history of *Dolomiaea*. Eight highly divergent regions (*rps*16*-trn*Q, *trn*C*-pet*N, *trn*E*-rpo*B, *trn*T*-trn*L*-trn*F, *psb*E*-pet*L, *ndh*F*-rpl*32*-trn*L, *rps*15*-ycf*1, and *ycf*1), along with a total of 51–61 simple sequence repeats (SSRs) were identified as valuable molecular markers for further species delimitation and population genetic studies. Phylogenetic analyses confirmed the evolutionary position of *Dolomiaea* as a clade within the subtribe Saussureinae, while revealing the discordance between the molecular phylogeny and morphological treatment. Our analysis also revealed that the plastid genes, *rpo*C2 and *ycf*1, which are rarely used in Asteraceae phylogenetic inference, exhibit great phylogenetic informativeness and promise in further phylogenetic studies of tribe Cardueae. Analysis for signatures of selection identified four genes that contain sites undergoing positive selection (*atp*A, *ndh*F, *rbc*L, and *ycf*4). These genes may play important roles in the adaptation of *Dolomiaea* to alpine environments. Our study constitutes the first investigation on the sequence and structural variation, phylogenetic utility and positive selection of plastomes of *Dolomiaea*, which will facilitate further studies of its taxonomy, evolution and conservation.

## Introduction

*Dolomiaea* DC. is a medicinally important genus of the sunflower family (Asteraceae) with approximately 15 species endemic to alpine habitats between 2800 and 4800 m in the Qinghai-Tibet Plateau (QTP) and adjacent areas (Ling, 1965; Shih, 1986; Wang et al., 2007; Wang X. et al., 2013; Chen and von Raab-Straube, 2013). Morphologically, *Dolomiaea* is similar to *Jurinea* Cass. (Asteraceae), and *Saussurea* DC. (Asteraceae), but is distinguished from these related genera by the presence of scabrid pappus bristles and naked alveolate receptacles without scales (Ling, 1965; Shih, 1986; Wang et al., 2007; Wang X. et al., 2013; Chen and von Raab-Straube, 2013). Two sections were recognized by Shih (1986) within the genus, including the primary group sect. *Dolomiaea* DC. with short, compact and apex-rounded style branches, and sect. *Vladimiria* (Iljin) Shih with divergent style-branches. Molecular phylogenetic analysis of *Dolomiaea* and related genera (Wang et al., 2007), along with a comprehensive systematic revision of the tribe Cardueae (Susanna and Garcia-Jacas, 2007, 2009), suggested the status of *Dolomiaea* as an independent clade within subtribe Saussureinae (tribe Cardueae), albeit *Dolomiaea* was absent in a more recent phylogenomic study (Herrando-Moraira et al., 2019).

*Dolomiaea* is commonly known as “Chuan Mu Xiang” in traditional Chinese medicine (Wei et al., 2014; Huang et al., 2019). Species of *Dolomiaea* are used medicinally for their significant bioactivities such as antioxidant (Chen et al., 2013), anti-inflammatory (Chen et al., 2017; Shi et al., 2017) and antimicrobial (Chen et al., 2015) properties, and are also rich resources of multiple chemical components such as sesquiterpenes (Chen et al., 2015), triterpenes (Fan et al., 2016), and phenylpropanoids (Wei et al., 2014). Despite significant medicinal value, genomic resources of *Dolomiaea* are lacking. Hence, taxonomic delimitation and the understanding of the evolutionary history of *Dolomiaea* remain hindered by insufficient information provided by Sanger sequencing of DNA markers.

Plastid genomes (plastomes) of photosynthetic plants are highly conserved in terms of gene content and structure (Ruhlman and Jansen, 2014). Typical plastome architecture is comprised of a quadripartite structure with two copies of an inverted repeat (IR) separated by large and small single copy (SSC) regions (LSC and SSC, respectively) (Palmer, 1991). In photosynthetic angiosperms, most plastomes contain approximately 80 protein-coding genes (PCGs) including photosynthetic genes, transcription and translation related genes, as well as some proteins related to other metabolic and synthesis processes, along with 30 transfer RNA (tRNA) genes and four ribosomal RNA (rRNA) genes (Ruhlman and Jansen, 2014). Due to the lack of recombination, usually uniparental inheritance and high copy numbers per cells (Wicke et al., 2011; Ruhlman and Jansen, 2014), whole plastome sequences have been extensively used in reconstructing the plant Tree of Life (e.g., Jansen et al., 2007; Moore et al., 2007; Ruhfel et al., 2014; Gitzendanner et al., 2018; Li et al., 2019). Comparative plastome studies provide the opportunity to explore sequence variation and the molecular evolutionary patterns associated with genome rearrangements (e.g., Knox, 2014; Weng et al., 2014; Rabah et al., 2019; Shrestha et al., 2019) as well as gene loss, duplication, and transfer events (e.g., Downie and Jansen, 2015; Wu and Chaw, 2016; Sun et al., 2017), while also detecting signatures of positive selection in plastid genes facilitating our understanding of plants adapting to extreme environments (e.g., alpine areas) (Bock et al., 2014; Jiang et al., 2018; Liu et al., 2018). Highly divergent regions and simple sequence repeats (SSRs) obtained from whole plastome sequence hold promise as efficient molecular markers implemented in species delimitation and population genetics (Zhang et al., 2017; Cui et al., 2019). Use of these markers as DNA barcodes for herbal medicine is promising for the authentication and identification of specimens for quality assurance (Sgamma et al., 2017; Wang et al., 2017; Kreuzer et al., 2019). In addition, although studies of phylogenetic utility of plastid genes have been widely documented (e.g., Logacheva et al., 2007; Neubig et al., 2009; Granados-Mendoza et al., 2013; Goncalves et al., 2019; Walker et al., 2019), phylogenetic informativeness of plastid genes in inferring intergeneric and infrageneric relationships within Asteraceae remains poorly understood.

In the present study, we sequenced and annotated plastomes of four species in *Dolomiaea*: *D. calophylla* Y. Ling, *D. denticulata* (Y. Ling) C. Shih, *Dolomiaea edulis* (Franch.) C. Shih, and *D. souliei* (Franch.) C. Shih. Among them, *D. calophylla* belongs to sect. *Dolomiaea*, and the other three species are members of Sect. *Vladimiria*. We analyzed our data in a comparative genomic framework within Cardueae to detect structural variation of the plastomes in this diverse tribe (with ca. 2400 species in 73 genera) (Susanna and Garcia-Jacas, 2009), as well as to identify highly divergent hotspots as molecular markers. Furthermore, a phylogenomic backbone of Cardueae was inferred to investigate the systematic position of *Dolomiaea* and examine phylogenetic informativeness of plastid PCGs (Townsend, 2007) in resolving the Cardueae phylogeny. Nucleotide substitution rates of plastid genes were also characterized to uncover possible mechanisms associated with *Dolomiaea* adapting to alpine environments. Overall, the current study is the first to investigate the sequence and structural variation, phylogenetic utility and positive selection of plastomes of the medicinally important genus *Dolomiaea*, while incorporating a phylogenomic framework.

## Materials and Methods

### Taxon Sampling and Sequencing

Fresh leaves of four *Dolomiaea* species representing both traditionally recognized sections were collected from the QTP and adjacent regions. The circumscription and the taxonomy of *Dolomiaea* were determined following the *Flora of China* or the Plant List database^1^. Voucher specimens were deposited in the Herbarium of Kunming Institute of Botany (KUN), Chinese Academy of Sciences. The voucher information is provided in Supplementary Table S1. For all species, total genomic DNA was extracted following the procedure of Plant Genomic DNA Kit (DP305) from Tiangen Biotech Co., Ltd. (Beijing, China). Paired-end Illumina libraries were constructed with the NEBNext Ultra DNA Library Prep Kit (New England Biolabs, Ipswich, MA, United States) according to the manufacturer’s protocol. A 500-bp DNA TruSeq Illumina (Illumina Inc., San Diego, CA, United States) sequencing library was constructed using 2.5–5.0 ng sonicated DNA as input and final quantifications were done using an Agilent 2100 Bioanalyzer (Agilent Technologies, Santa Clara, CA, United States) and real-time quantitative PCR. Libraries were multiplexed and sequenced using a 2 × 150 bp run on an Illumina HiSeq 2500 platform at Novogene Co., Ltd. in Kunming, Yunnan, China.

### Plastome Assembly and Annotation

Raw sequence reads were cleaned using Trimmomatic v.0.36 (Bolger et al., 2014) by removing duplicate reads and trimming adapter-contaminated reads. Remaining high-quality reads were assembled *de novo* into contigs in NOVOPlasty v.2.7.2 (Dierckxsens et al., 2017) using a seed-and-extend algorithm with the plastome sequence of *Saussurea japonica* (Thunb.) DC. (GenBank accession: MK953481.1) as the seed input due to a close relationship of *Dolomiaea* with *Saussurea* (Wang et al., 2007). Other parameters were left at default settings (see NOVOPlasty manual). Assembled plastomes were annotated with the “Annotate from source” tool in Geneious v.9.0.5 (Kearse et al., 2012) using a BLAST-like algorithm to search for annotations in the specified “Source” folder (Kearse et al., 2012) which included all the publicly available plastomes of Cardueae (see Supplementary Table S1 for GenBank accession numbers). Start/stop codons and intron/exon boundaries were manually inspected, and the tRNA genes were identified with tRNAscan-SE (Lowe and Eddy, 1997) as implemented in GeSeq (Tillich et al., 2017). Graphical maps of the circular plastomes were visualized with OGDRAW (Lohse et al., 2013).

### Comparative Plastome Analysis

To detect the presence of IR expansion or contraction among four plastomes of *Dolomiaea*, we used *S. japonica* as reference and visualized the borders of the large single-copy (LSC), small single-copy (SSC), and IR regions among the five species in Irscope (Amiryousefi et al., 2018). We downloaded the publicly available plastomes of eight species from different genera of Cardueae (i.e., *Arctium lappa* L., *Atractylodes chinensis* (Bunge) DC., *Carthamus tinctorius* L., *Centaurea diffusa* Lam., *Cirsium arvense* (L.) Scop., *Cynara humilis* L., *S. japonica*, and *Silybum marianum* (L.) Gaertn.; GenBank accession numbers are provided in Supplementary Table S1). The percentage of sequence identity was plotted with nine species representing the nine genera of Cardueae using the mVISTA program (Frazer et al., 2004) with LAGAN mode (Brudno et al., 2003).

### Identification of Molecular Markers

Simple sequence repeats across the plastomes of four *Dolomiaea* species were detected using the MISA-web application (Beier et al., 2017)^2^. Thresholds for a minimum number of repeat units were established as follows: ten for mono-nucleotide, five for di-nucleotide, four for tri-nucleotide, and three for tetra-nucleotide, penta-nucleotide, or hexa-nucleotide SSR.

To observe the sequence divergence and determine highly divergent hotspots of Cardueae plastomes, 22 publicly available plastomes were downloaded from the National Center for Biotechnology Information (NCBI) database (Supplementary Table S1). All plastome sequences of Cardueae with only one IR region included were aligned using MAFFT v.7.22 (Katoh and Standley, 2013) under “–auto” strategy. Nucleotide diversity (π) was calculated by sliding window analysis conducted in DnaSP v.6.11.01 (Rozas et al., 2017). The step size was set to 200 bp, with a 600 bp window.

### Phylogenetic Analyses

For phylogenetic analyses, 28 taxa were sampled (Supplementary Table S1), including 26 taxa of Cardueae (four newly sequenced and 22 downloaded from NCBI representing all available genera of Cardueae) and two outgroup taxa of tribe Cichorieae (*Taraxacum officinale* F. H.Wigg. and *Lactuca sativa* L.) based on the availability and previous study (Fu et al., 2016). All 79 PCGs were extracted in PhyloSuite v.1.1.16 (Zhang D. et al., 2019). Initial sequences were aligned using the codon-aware program MACSE v. 2.03 (Ranwez et al., 2018), which preserves reading frame and allows incorporation of sequencing errors or sequences with frameshifts. These aligned sequences were then concatenated into a supermatrix using PhyloSuite, and the number of parsimony informative sites was calculated using MEGA X (Kumar et al., 2018).

Both maximum-likelihood (ML) and Bayesian inference (BI) analyses were conducted. For the ML analysis, RAxML v.7.4.2 (Stamatakis, 2014) was used under the general time reversible model of nucleotide substitution with the gamma model of rate heterogeneity (GTR + G) as suggested (see RAxML manual). Twenty independent ML searches and 1000 rapid Bootstrap replicates were executed with “-f a -x 12345 -# 1000” option in RAxML analysis. Bayesian inference was conducted in MrBayes v.3.2.3 (Huelsenbeck and Ronquist, 2001) with the optimal model (GTR + G + I) calculated by jModelTest v.2.1.10 (Darriba et al., 2012) under the Bayesian information criterion (BIC). Two runs were conducted in parallel with four Markov chains (one cold and three heated), with each running for 2,000,000 generations from a random tree and sampled every 200 generations. Convergence was checked by examining the average standard deviation of split frequencies (ASDF). After ASDF reached < 0.01, the initial 25% of the sampled data were discarded as burn-in, and the remaining trees were used to construct a majority-rule consensus tree and calculate the posterior probability. We also conducted partitioned ML and BI analyses. PartitionFinder v.1.0.1 (Lanfear et al., 2012) was implemented to determine optimal partitioning scheme and evolutionary model selection under the BIC. The best-fit evolutionary models of partitioning subsets were used in the partitioned phylogenetic inferences. Other settings were consistent with the unpartitioned analyses. The final phylogenetic results were viewed using FigTree v.1.6.1 (Rambaut and Drummond, 2010).

### Phylogenetic Informativeness of Plastid Genes

The PhyDesign web application^3^ was used to estimate the phylogenetic informativeness profiles for the 79 PCGs using the HyPhy substitution rates algorithm for DNA sequences with the default settings (Pond et al., 2005; Townsend, 2007; Lopez-Giraldez and Townsend, 2011). The inferred tree from the concatenated ML analysis in RAxML was used as an input tree to reconstruct a relative-time ultrametric tree in the *dnamlk* program in PHYLIP (Felsenstein, 1989). The converted relative-time ultrametric tree and alignment of 79 PCGs partitioned by genes were used as input files in PhyDesign to calculate phylogenetic informativeness.

### Positive Selection on Plastid Genes

We used the branch-site model in codeml program of the package PAML v.4.9h (Yang, 2007) to identify plastid genes under positive selection in *Dolomiaea*. The 79 PCGs used in the phylogenetic analysis were tested. The ML tree generated via RAxML was used as a constraint topology, and the clade formed by *Dolomiaea* was set as a foreground branch. Likelihood ratio test (LRT) was conducted to compare a model allowing positive selection (the ratio of non-synonymous to synonymous substitutions: ω > 1) acting on a site in the foreground branch with a null model where the site may have undergone neutral evolution (ω = 1) or purifying selection (ω < 1). A Chi square *p*-value smaller than 0.05 were used as cutoff of significance. The Bayes Empirical Bayes (BEB) inference (Yang et al., 2005) was then implemented in site models M2a and M8 to estimate the posterior probabilities and positive selection pressures of the selected genes.

## Results

### Genome Assembly and Plastome Features

**FIGURE 1:**
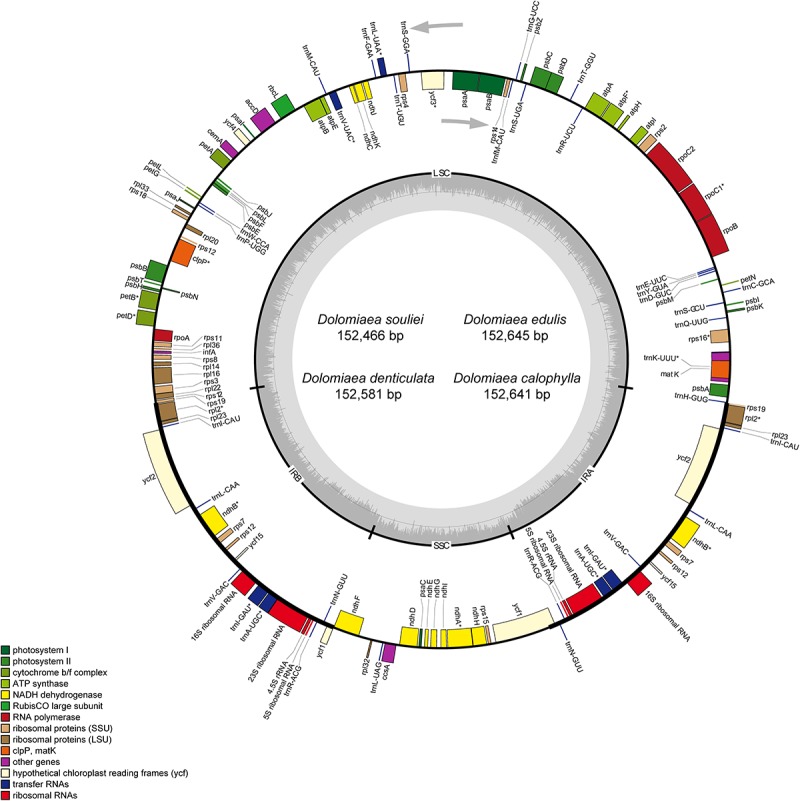
Map of four newly sequenced plastomes of *Dolomiaea*. Genes outside the circle are transcribed in a counter-clockwise direction, whereas those inside the circle are transcribed in a clockwise direction. The dark gray area in the inner circle indicates GC content and the thick line shows the extent of different regions. Different colors for genes indicate different functional groups. LSC: large single copy; SSC: small single copy; IR: inverted repeat.

After Illumina sequencing, a total of 13,571,156–18,476,472 pair-end clean reads were obtained for each species (Table 1). The mean sequencing coverage ranged from 412 × (*D. edulis*) to 1,264 × (*D. denticulata*). All four plastomes displayed the typical quadripartite structure composed of a large single copy (LSC), a SSC, and two IRs (IRa and IRb). The length of the four plastomes ranged from 152,466 bp in *D. souliei* to 152,645 bp in *D. edulis* (Table 1). All the plastomes contain 79 PCGs, four rRNAs and 30 tRNAs arranged in the same gene order (Figure 1). A total of 18 genes (including 11 PCGs and seven tRNA genes) had introns, with 15 genes having one intron and three genes having two introns. The *rps*12 gene was found to be trans-spliced in all plastomes, with one of its exons located in the LSC region and the other duplicated in the IR (Figure 1).

**TABLE 1:** Assembly information of newly sequenced plastomes of four *Dolomiaea* species.

**Species**	***D. calophylla***	***D. denticulata***	***D. edulis***	***D. souliei***
Total reads	18,476,472	13,571,156	16,158,610	15,236,864
Assembled reads	438,732	529,500	271,020	238,390
Average coverage	842	1,264	412	480
Plastome size (bp)	152,641	152,581	152,645	152,466
LSC length (bp)	83,675	83,533	83,631	83,449
IR length (bp)	25,196	25,219	25,205	25,190
SSC length (bp)	18,574	18,610	18,604	18,637
GC content (%)	37.7	37.6	37.7	37.7

**FIGURE 2:**
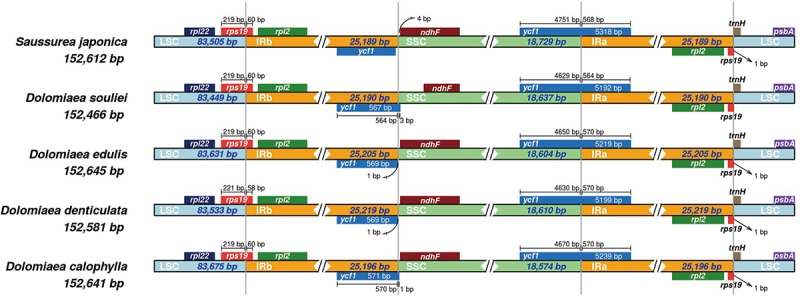
Comparison of IR-SC border positions across plastomes of four *Dolomiaea* species and *Saussurea japonica*. Genes are denoted by colored boxes. The gaps between the genes and the boundaries are indicated by the base lengths (bp).

The IR regions were highly consistent in plastomes of *Dolomiaea*, comprising a length of 25,190–25,219 base pairs and including 17 genes (six PCGs, seven tRNA genes, and four rRNA genes). Two pseudogenes, *rps*19 and *ycf*1, were identified. The IRb/LSC junction was located within the *rps*19 gene in all plastomes, resulting in the presence of a part of the *rps*19 gene in the IRa. Similarly, the IRa/SSC boundary positions in all species were located in the *ycf*1 gene, with part of this gene duplicated in the IRb (Figure 2).

**FIGURE 3:**
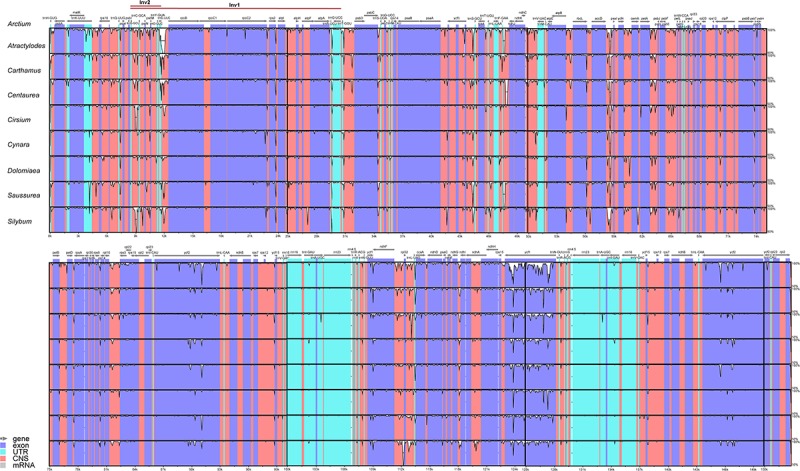
Visualization of the alignments of nine plastomes representing nine genera of Cardueae using mVISTA, with *Arctium lappa* as the reference. The gray arrows above the alignment indicate genes. Different colors represent different regions (coding and non-coding). The horizontal axis indicates the coordinates within the chloroplast genome. The vertical scale represents the percentage of identity, ranging from 50% to 100%.

To investigate the sequence divergence of plastomes, the percentage of sequence identity was plotted for nine representative species from different genera of Cardueae using the mVISTA program (Frazer et al., 2004) with *A. lappa* as the reference. High similarity was detected among those nine taxa, suggesting that plastome sequences are conserved in Cardueae. The IR regions were found to be more conserved than single-copy regions, and coding regions are more conserved than non-coding regions (Figure 3). All analyzed plastomes possessed the ∼20 and ∼3 kb inversions (Inv1 and Inv2), which have been widely detected in plastomes of Asteraceae (e.g., Kim et al., 2005; Liu et al., 2013; Walker et al., 2014; Zhang X. et al., 2019). The Inv2, located between the *trn*S*-*GCU and *trn*E*-*UUC genes, was nested within the Inv1, located between the *trn*G*-*UCC and *trn*S*-*GCU genes (Figure 3).

### Molecular Markers

**FIGURE 4:**
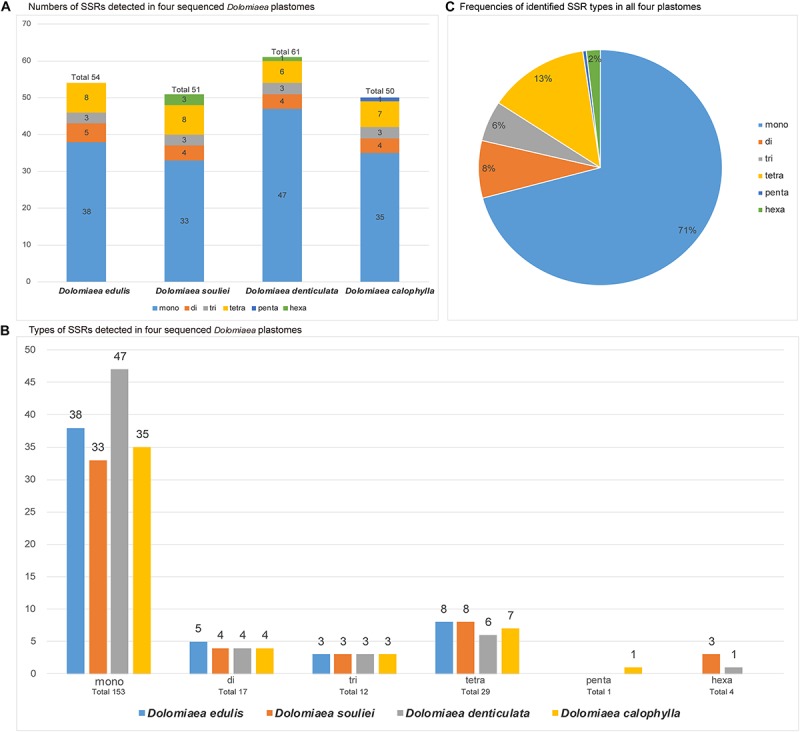
Comparison of simple sequence repeats (SSRs) among four plastomes. **(A)** Numbers of SSRs detected in the four newly sequenced Cardueae plastomes. **(B)** Types of SSR types detected in all four plastomes. **(C)** Frequencies of identified SSR types in all four plastomes.

**FIGURE 5:**
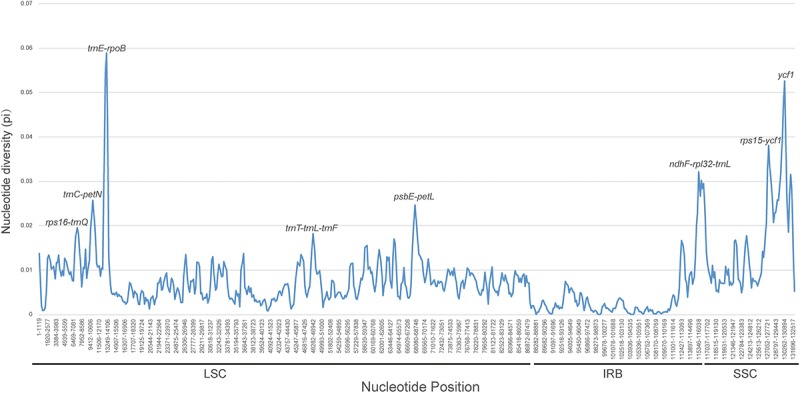
Sliding window analysis of nucleotide diversity (π) along the whole plastome for the 26 aligned plastomes of Cardueae with one IR copy included. Genes underlying peaks of nucleotide diversity are labeled.

The counts and types of SSRs were generally similar between the newly sequenced plastomes (Figure 4). The number of SSRs ranged from 51 (*D. souliei*) to 61 (*D. denticulata*) (Figure 4A). The types of SSRs in each plastome were quite similar (Figure 4A). In all four plastomes, the total number of SSRs for mono-nucleotide, di-nucleotide, tri-nucleotide, tetra-nucleotide, penta-nucleotide, and hexa-nucleotide were 153, 17, 12, 29, 1, and 4 (Figures 4B,C), respectively. Nucleotide diversity (π) was calculated by sliding window analysis to observe the sequence divergence and determine highly divergent hotspots. The single-copy regions have higher proportions of variable sites than in the IR regions (Figure 5). By scanning for variation throughout the whole plastome, we determined eight highly divergent regions, namely *rps*16*-trn*Q, *trn*C*-pet*N, *trn*E*-rpo*B, *trn*T*-trn*L*-trn*F, *psb*E*-pet*L, *ndh*F*-rpl*32*-trn*L, *rps*15*-ycf*1, and *ycf*1 (Figure 5).

### Phylogenetic Relationships

**FIGURE 6:**
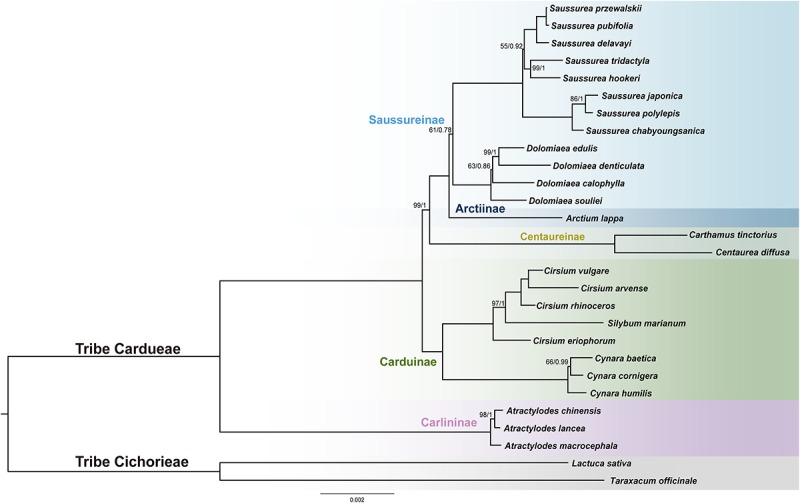
Phylogenetic reconstruction of Cardueae from maximum likelihood (ML) and Bayesian inference (BI) analyses using concatenated 79 protein-coding regions. The ML tree is shown. Maximum likelihood bootstrap values (BS) and posterior probabilities (PP) are shown at nodes. Branches with no values listed have 100% BS and PP of 1.00.

The final alignment of the 79-PCGs, 28-species data set consisted of 69,546 bp containing 2,485 parsimony-informative sites. PartitionFinder identified 11 subsets, and the best-fit substitution models of partitioning subsets were provided in Supplementary Table S2. Both unpartitioned and partitioned ML and BI analyses yielded identical tree topology [Figure 6 and Supplementary Figure S1; bootstrap values (BS) and posterior probabilities (PP) are depicted in ML tree]. Four species of *Dolomiaea* formed a clade with 100% support (BS = 100, PP = 1.0). *Dolomiaea souliei* is the sister species to a clade formed by *D. calophylla* and *D. edulis* + *D. demticulata*. Our results do not support the two morphological sections, i.e., sect. *Dolomiaea* and sect. *Vladimiria*, identified by Shih (1986) via long and acute vs. short and round style, as *D. calophylla* is nested within the other *Dolomiaea* species. *Dolomiaea* was revealed as a sister to *Saussurea* albeit with low support (BS = 61, PP = 0.78). The two genera constitute the subtribe Saussureinae in our analysis and are sister to subtribe Arctiinae. In tribe Cardueae, four subtribes included (Carlininae, Caduinae, Centaureinae, and Saussureinae) were monophyletic. Carlininae was the earliest-diverging subtribe, Caduinae was next, and Centaureinae was sister to Arctiinae + Saussureinae. Most genera included were supported as monophyletic except *Cirsium* (Asteraceae), showing a nested position with *Silybum* (Asteraceae).

### Phylogenetic Informativeness

**FIGURE 7:**
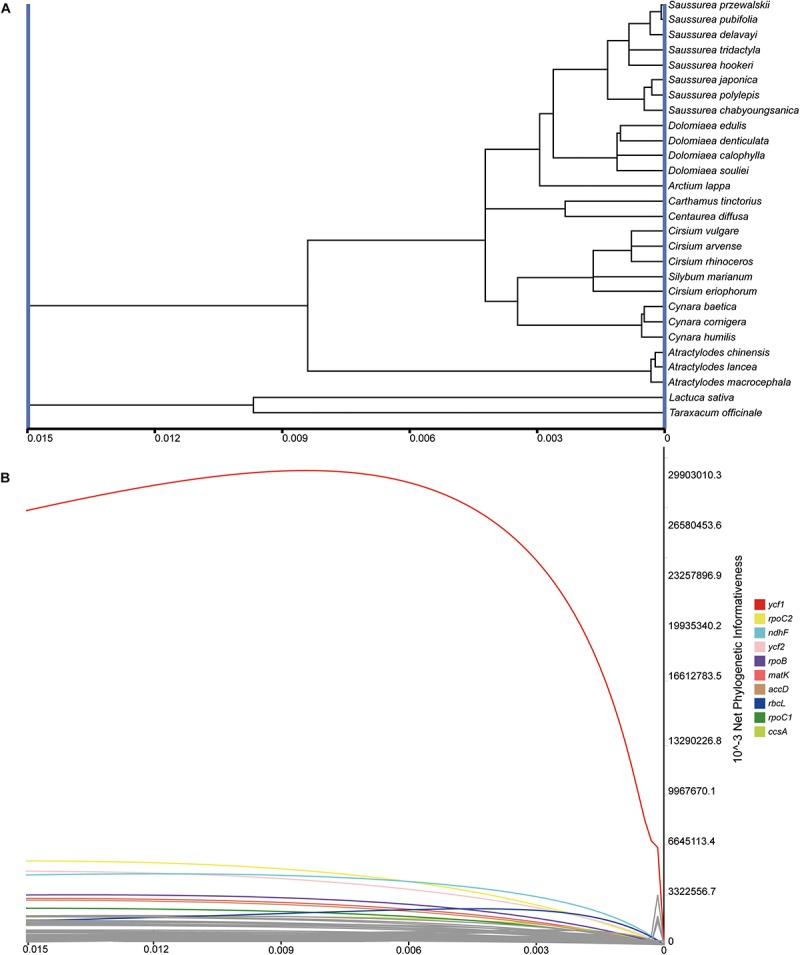
Phylogenetic net informativeness profiles of 79 PCGs of Cardueae estimated in PhyDesign (Lopez-Giraldez and Townsend, 2011). **(A)** The ultramteric tree of Cardueae converted in the *dnamlk* program in PHYLIP (Felsenstein, 1989). **(B)** Net phylogenetic informativeness profiles for 79 PCGs. Ten genes with the greatest informativeness are color-coded and indicated at the right. *X*- and *Y*-axes represent relative-time and net phylogenetic informativeness, respectively.

The net and per-site phylogenetic informativeness (PI) profiles for the 79 PCGs were measured using PhyDesign (Figure 7 and Supplementary Table S3). The *ycf*1 gene had the highest net phylogenetic informativeness among all PCGs, followed by *rpo*C2, *ndh*F and *ycf*2. Genes with high net PI were also genes with longer length, suggesting a large contribution of gene length to phylogenetic informativeness. For the per-site PI, *ycf*1 also performed best among PCGs, followed by *rps*16 and *ndh*F (Supplementary Table S3). The long gene *ycf*2 had a comparatively low per-site PI possibly due to a low frequency of rapidly evolving sites (Shrestha et al., 2019). Relatively conservative genes with less net PI were primarily associated with photosynthesis and were shorter in length (<200 bp).

### Positive Selection

Analyses of selection were conducted to identify signals of episodic selection occurring on PCGs along a specified branch (the clade formed by *Dolomiaea* in our analysis). Comparison against a branch−site model allowing positive selection along specified branches (Model A) with a null model (Model A_null_) allowing neutral evolution and negative selection was employed (Yang et al., 2005). We observed signatures of positive selection in four PCGs, *atp*A (3 sites), *ndh*F (5 sites), *rbc*L (4 sites), and *ycf*4 (1 sites) at a significance level of 0.05 (Table 2).

**TABLE 2:** Positively selected genes and sites detected in the plastomes of *Dolomiaea* species.

**Gene name**	**Positive sites**	**LRT *p*-value**
*rbc*L	37 E 0.968*, 102 S 0.992*, 235 A 0.972*, 479 M 0.997*	0.0035
*atp*A	306 S 0.994*, 498 M 0.975*	0.0372
*ndh*F	129 S 0.957*, 224 V 0.978*, 494 V 0.968*, 522 N 0.484, 639 I 0.997*, 688 N 0.961*	0.0136
*ycf*4	151 A 0.952*	0.0478

## Discussion

### Plastome Features

In this study, we assembled and analyzed four complete plastomes of *Dolomiaea* species. All four plastomes share the gene content and structure of most Asteraceae plastomes, and are similar to other angiosperm plastomes. The expansion/contraction of IR regions has been demonstrated to be a significant contribution to substantial variation in plastome size (Ruhlman and Jansen, 2014). Occurrence of IR expansion/contraction is common in angiosperm plastomes and has been documented in several lineages, such as *Pelargonium* (Weng et al., 2017), Trochodendraceae (Sun et al., 2013), *Plantago* (Zhu et al., 2016), and *Passiflora* (Rabah et al., 2019). In the present study, however, no significant IR length variation was found among *Dolomiaea* plastomes and with plastomes of its sister genus (*Saussurea*) (Zhang X. et al., 2019), indicating the conservative nature of plastome evolutionary history of *Dolomiaea*.

The percentage of sequence identity show high similarity among nine plastomes of Cardueae, suggesting that plastome sequences are conserved in this tribe despite being one of the most diverse tribes in Asteraceae. The two large inversions (Inv1 and Inv2 in Figure 3) in LSC are detected in all analyzed plastomes. These two large inversions are commonly found in plastomes of Asteraceae, e.g., *Lactuca* (Kim et al., 2005), *Artemisia* (Liu et al., 2013), *Lasthenia* (Walker et al., 2014), *Mikania* (Su et al., 2018), and *Saussurea* (Zhang X. et al., 2019), implying that such genome organization may reflect common evolutionary history in the family. Although uncommon, the structural variation of inversions has been discovered in many other angiosperm lineages, such as the ∼49 kb inversion discovered in the early diverging eudicot family Circaeasteraceae (Sun et al., 2017); the ∼78 kb inversion present in the Fabaceae subtribe Phaseolinae (Bruneau et al., 1990), the ∼36 kb inversion shared by all core genistoid legumes (Martin et al., 2014); and ∼20 kb inversion occurring in Styracaceae of Ericales (Yan et al., 2017, 2018). These inversions are considered highly valuable in phylogenetics due to their rarity, easily determined homology and easily inferred state polarity (Jansen et al., 2008).

### Molecular Markers

Species of *Dolomiaea* are commonly used in medicine for their significant bioactivities and multiple chemical components (Chen et al., 2015). However, the lack of genomic resources of *Dolomiaea* has hindered our deep exploration of its taxonomy, genetics and conservation. Assembling plastome sequences not only provides opportunity for illustrating the plastome evolution, but also generates valuable genetic resources such as SSRs and highly divergent regions helpful in further population genetics and taxonomic studies of *Dolomiaea*. In this study, the number of SSRs identified within *Dolomiaea* plastomes ranges from 51 to 61. The mononucleotide repeats with A and T repeat units are found to be the most abundant, which also were reported in other Asteraceae genera, e.g., *Jacobaea* (Doorduin et al., 2011), *Artemisia* (Shen et al., 2017), *Dendrosenecio* (Gichira et al., 2019) and *Saussurea* (Zhang X. et al., 2019), and other lineages such as *Nymphoides* and *Menyanthes* (Menyanthaceae) (Njuguna et al., 2019), *Sinadoxa* (Adoxaceae) (Wang et al., 2016), and *Actinidia* (Actinidiaceae) (Yao et al., 2015). Eight highly divergent regions (*rps*16*-trn*Q, *trn*C*-pet*N, *trn*E*-rpo*B, *trn*T*-trn*L*-trn*F, *psb*E*-pet*L, *ndh*F*-rpl*32*-trn*L, *rps*15*-ycf*1, and *ycf*1) are identified by scanning for variation throughout the whole plastomes. Most of the divergent regions are overlapping with those determined in plastomes of *Saussurea* (Zhang X. et al., 2019), reflecting their close affinity. Additionally, most of them, i.e., *rps*16*-trn*Q, *trn*L*-trn*F, *psb*E*-pet*L, *rpl*32*-trn*L, and *ycf*1, have been previously reported as rapidly diverging loci and are broadly used for reconstructing phylogenies (Neubig et al., 2009; Prince, 2015; Gao et al., 2018; Su et al., 2018). For the herbal medicinal genus *Dolomiaea*, these genetic resources as molecular markers can serve as promising DNA barcoding in the authentication of specimens for medicinal quality assurance.

### Phylogenetic Relationships and Phylogenetic Informativeness of Plastid Genes

Our phylogenetic analyses show a sister relationship between Saussureinae and Arctiinae, which is consistent with phylogenetic results of Herrando-Moraira et al. (2019) using nuclear data, but inconsistent with their plastome data results. We speculate this inconsistency likely results from the difference of sequencing strategy between the present study with the study of Herrando-Moraira et al. (2019), in which they recovered plastome sequences using a Hyb-Seq approach. Only targeted DNA regions are enriched in Hyb-Seq approaches using specific probes or “baits” (Herrando-Moraira et al., 2019), and thus plastome sequences generated from target enrichment are likely incomplete. Overall, the phylogenetic discordance from different data sets, along with weakly supported resolution of some relationships, suggests a possible complex evolutionary history involving hybridization and incomplete lineage sorting, and further investigation using more advanced methods, as well as a wider species sampling, are necessary.

The present phylogenomic framework confirms the systematic position of *Dolomiaea* as a monophyletic group within subtribe Saussureinae. Phylogenetic relationships within the Saussureinae on a large scale remain ambiguous, mainly because of the high number of small segregates separated from the large genera *Saussurea* or *Jurinea* using molecular data. As proposed, there may be three genera (*Dolomiaea*, *Jurinea*, and *Saussurea*) that constitute the Saussureinae (Susanna and Garcia-Jacas, 2009). Our analysis resolves *Dolomiaea* as a sister to *Saussurea* with weak support, indicating the relationship is not very robust, and future studies adding *Jurinea* species are needed. The early diverging position of *D. edulis* was consistent with the study of Wang et al. (2007) using nuclear rDNA (ITS) and plastid *trn*L*-*F + *psb*A*-trn*H regions. Furthermore, we reveal the discordance between the molecular phylogeny and morphological treatment of *Dolomiaea*, which may suggest the infrageneric classification of the circumscribed genus needs further revision.

As one of the largest tribes in Asteraceae, Cardueae is also one of the most complicated because of great morphological diversity and because it comprises some of the largest genera of the family, such as *Cirsium* Mill. (250 spp.), *Centaurea* L. (400 spp.), *Cousinia* Cass. (600 spp.), and *Saussurea* DC. (300 spp.) (Susanna and Garcia-Jacas, 2009). Although some progress in reconstructing phylogenetic relationships within Cardueae has been made using plastid markers, no study has yet to examine the phylogenetic utility of plastid genes in this tribe. A major bottleneck in molecular phylogenetic studies of recently diverged lineages is the insufficient resolution provided by loci with limited phylogenetic signal. Therefore, identifying plastid markers with high phylogenetic informativeness will aid efforts in resolving complex phylogenetic relationships at the species-level, as well as in plant barcoding. All three genes (*mat*K, *ndh*F and *rbc*L) widely used in phylogenetic studies of Cardueae (Susanna et al., 2006; Barres et al., 2013; Wang Y.J. et al., 2013; Fu et al., 2016), showed a high value of phylogenetic informativeness. Nonetheless, *ycf*1 and *rpo*C2 exhibited high phylogenetic informativeness values and relatively long gene length, yet have not been employed in phylogenetic analyses of Cardueae. Previous studies have indicated the usefulness of *ycf*1 in plant phylogenetics, such as orchids (Neubig et al., 2009), Annonaceae (Neubig and Abbott, 2010), and Amaryllidaceae (García et al., 2014). A recent study of Walker et al. (2019) revealed the good performance of *ycf*1 and *rpo*C2 in the phylogenetic reconstruction of the angiosperm phylogeny, which is also supported by our current results. Hence, we advocate the usefulness of *ycf*1 and *rpo*C2 as promising plastid markers for inferring evolutionary relationships of Cardueae.

Given primer universality as an important criterion for an ideal DNA marker in phylogenetics and plant barcoding (Dong et al., 2015), primer design is a possible obstacle to the application of *ycf*1 and *rpo*C2 in Sanger sequencing. Our study presents a routine procedure for marker selection at the molecular level. Taking the availability of primers into account is essential for more practical evaluation of the phylogenetic informativeness of a marker in the further studies.

### Signature of Positive Selection on Plastid Genes

Our study reveals that natural selection can target different functional groups of plastid genes and support the possible involvement of plastid genes in plant adaptation to alpine environments. *Dolomiaea* species are distributed mainly in high-altitude environments in the QTP, which pose a variety of stress factors including low temperature, strong UV radiation, low oxygen level, and capricious climate (Zhang T. et al., 2019). Genes under positive selection may play important roles in the adaptation of *Dolomiaea* to the harsh environments.

Among the 79 PCGs within *Dolomiaea*, four (*atp*A, *ndh*F, *rbc*L, and *ycf*4) show evidence of undergoing positive selection. The *atp*A gene, encoding the alpha subunit of ATP synthase, plays a vital role in energy-transduction during photosynthesis (Walker, 2013). Accelerated rates of evolution in *atp*A may promote the specialization of ATP synthases enhancing the efficiency of energy-transduction in photosynthesis at high-altitude environments with low CO_2_ concentration. The *atpF* gene encodes the NADH dehydrogenase unit which is involved in cyclic electron flow around photosystem I essential for photosynthesis (Munekage et al., 2004). Positive selection of *ndh*A is possibly related to the protection of *Dolomiaea* species from strong light damage in higher altitudes. Positive selection might be acting on the *rbc*L gene which encodes the large subunit of the photosynthetic enzyme Rubisco and is related to plants adaptation to low temperature, drought and carbon dioxide concentration. Selection on this gene has also been observed in various plant groups such as *Panax* (Araliaceae) (Jiang et al., 2018), *Silene* (Caryophyllaceae), and *Potamogeton* (Potamogetonaceae) (Iida et al., 2009), and is particularly prevalent following the evolution of C_4_ photosynthesis (Piot et al., 2018).

## Conclusion

Previous phylogenetic analysis using fragment DNAs suggested *Dolomiaea* as a clade within subtribe Saussureinae (Wang et al., 2007), however the most recent phylogenomic study did not include any samples of *Dolomiaea* (Herrando-Moraira et al., 2019). Our phylogenomic framework based on whole plastomes confirm the systematic position of the genus, while revealing the discordance between the molecular phylogeny and morphological treatment suggesting the infrageneric classification of the circumscribed genus needs further revision. Our analysis also reveals that the rarely used plastid genes in Asteraceae phylogenetic inference, *rpo*C2 and *ycf*1, exhibit great phylogenetic informativeness and substantial promise in further phylogenetic studies of Cardueae. By comparative analyses, we detect the sequence and structural conservatism of plastomes indicating *Dolomiaea* may have a recently diverged history, and/or slow mutation rates of plastid genes. As a medicinally important genus endemic to the QTP, species identification and authentication, as well as resource conservation, are of importance for medicinal quality assurance. The highly divergent plastome regions determined in this study will be helpful in taxonomy and population genetics of *Dolomiaea.* Furthermore, given the lack of knowledge about the underlying mechanisms for plants adapting to alpine environments, the signature of positive selection on plastid genes identified here provides new insights into the role plastomes play in adaptive evolution.

## Data Availability Statement

All newly sequenced plastome sequences have been deposited in NCBI. Accession numbers can be found in Supplementary Table S1.

## Author Contributions

HW and HS conceived and designed the study. JS and XZ performed *de novo* assembly, genome annotation, phylogenetic, and other analyses. JS, XZ, JL, HW, and HS drafted the manuscript. XZ and TD collected the leaf materials. JS and HZ performed the experiments. All authors discussed the results and helped shape the research, analyses, and final manuscript.

## Conflict of Interest

The authors declare that the research was conducted in the absence of any commercial or financial relationships that could be construed as a potential conflict of interest.

## Footnotes

1 http://www.theplantlist.org/

2 https://webblast.ipk-gatersleben.de/misa/

3 http://phydesign.townsend.yale.edu/

## Supplementary Material

FIGURE S1 Phylogenetic reconstruction of Cardueae from partitioned maximum likelihood (ML) and Bayesian inference (BI) analyses using concatenated 79 protein-coding regions. The ML tree is shown. Maximum likelihood bootstrap values (BS) and posterior probabilities (PP) are shown at nodes. Branches with no values listed have 100% BS and PP of 1.00.

TABLE S1 Taxa included in the present study. NCBI accession numbers and voucher specimens’ information are provided for newly sequenced plastomes.

TABLE S2 The best-fit evolutionary models of 11 partitioning subsets calculated by PartitionFinder. # Sites: the number of sites of the partitioning subsets.

TABLE S3 Phylogenetic informativeness profiles for 79 plastid protein-coding genes. Included are number of sites for which substitution rates were calculated (#Rates), net phylogenetic informativeness at epochs: 0–0.015 (Net PI) and per-site phylogenetic informativeness (Per-site PI). Ten genes with the most net informativeness are colored in red.
