# Supplementary figures and images for: Plastome Evolution in Dolomiaea (Asteraceae, Cardueae) Using Phylogenomic and Comparative Analyses
Source: Front Plant Sci. 2020 Apr 15;11:376. doi: 10.3389/fpls.2020.00376 (PMC7174903; doi:10.3389/fpls.2020.00376)

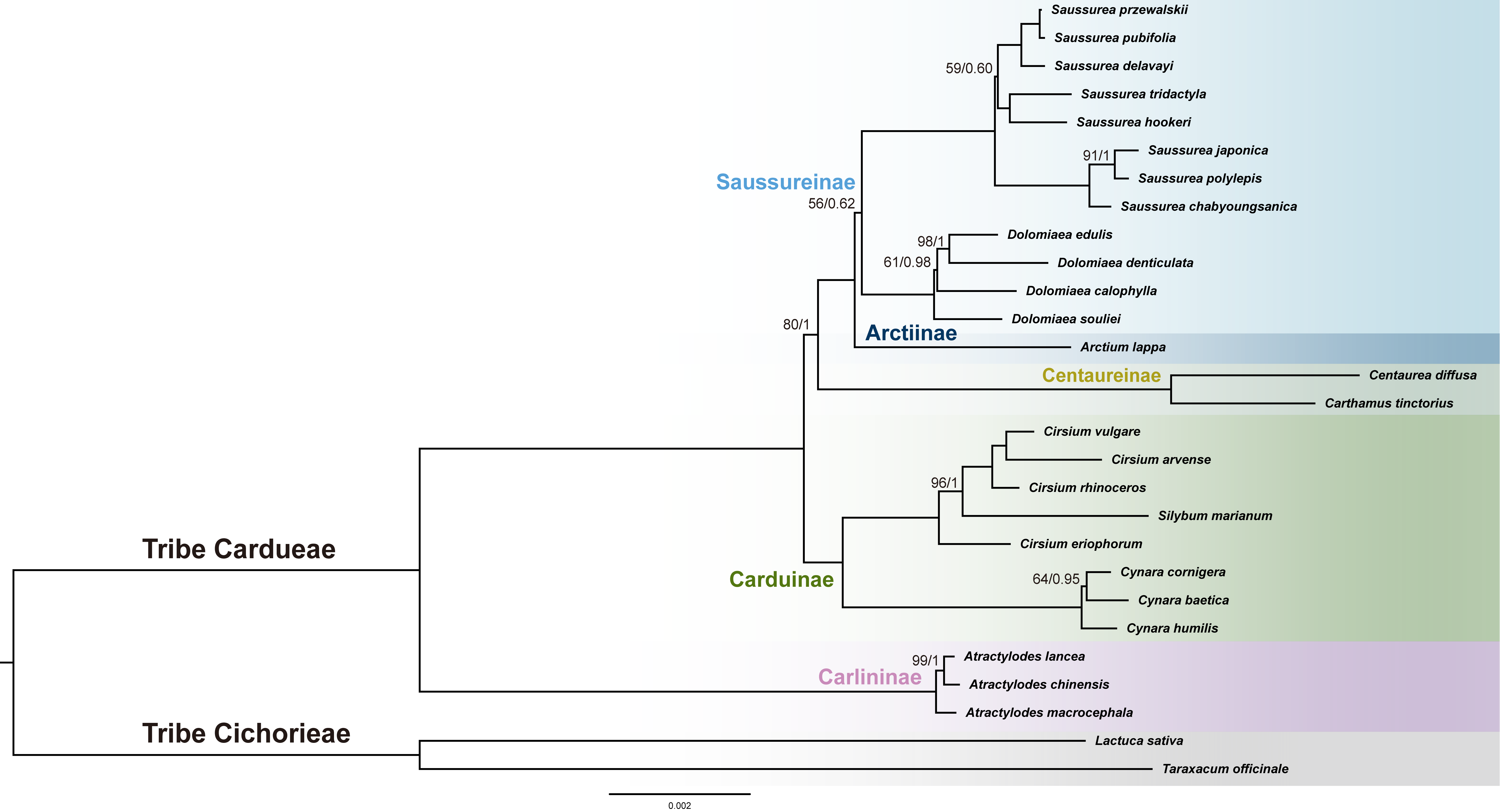

Supplement: FIGURE S1 — Phylogenetic reconstruction of Cardueae from partitioned maximum likelihood (ML) and Bayesian inference (BI) analyses using concatenated 79 protein-coding regions. The ML tree is shown. Maximum likelihood bootstrap values (BS) and posterior probabilities (PP) are shown at nodes. Branches with no values listed have 100% BS and PP of 1.00. [file Image_1.TIF]
