# Supplementary material for: Plastome Evolution in Dolomiaea (Asteraceae, Cardueae) Using Phylogenomic and Comparative Analyses
Source: Front Plant Sci. 2020 Apr 15;11:376. doi: 10.3389/fpls.2020.00376 (PMC7174903; doi:10.3389/fpls.2020.00376)
Supplement: TABLE S1 — Taxa included in the present study. NCBI accession numbers and voucher specimens’ information are provided for newly sequenced plastomes. [file Table_1.DOCX]

**Table S1** Taxa included in the present study. NCBI accession numbers and voucher specimens’ information are provided for newly sequenced plastomes

| **Tribe** | **Subtribe** | **Species** | **NCBI accession numbers** | **Length (bp)** | **GC content (%)** | **Voucher specimens** |
| --- | --- | --- | --- | --- | --- | --- |
| Cardueae | Saussureinae | *Dolomiaea calophylla* | MT128668 | 152,641 | 37.7 | ZJW5631 |
| Cardueae | Saussureinae | *Dolomiaea denticulata* | MT128669 | 152,581 | 37.6 | SunH-07ZX-3416 |
| Cardueae | Saussureinae | *Dolomiaea edulis* | MT128670 | 152,645 | 37.7 | ZJW5130 |
| Cardueae | Saussureinae | *Dolomiaea souliei* | MT128671 | 152,466 | 37.7 | FSC-323 |
| Cardueae | Saussureinae | *Saussurea chabyoungsanica* | KX622799.1 | 152,446 | 37.7 | - |
| Cardueae | Saussureinae | *Saussurea delavayi* | MK953476.1 | 152,254 | 37.7 | - |
| Cardueae | Saussureinae | *Saussurea hookeri* | MK952740.1 | 152,461 | 37.7 | - |
| Cardueae | Saussureinae | *Saussurea japonica* | MK953481.1 | 152,612 | 37.7 | - |
| Cardueae | Saussureinae | *Saussurea polylepis* | MF695711.1 | 152,488 | 37.6 | - |
| Cardueae | Saussureinae | *Saussurea przewalskii* | MK953475.1 | 152,658 | 37.7 | - |
| Cardueae | Saussureinae | *Saussurea pubifolia* | MK953467.1 | 152,622 | 37.7 | - |
| Cardueae | Saussureinae | *Saussurea tridactyla* | MK953472.1 | 151,474 | 37.7 | - |
| Cardueae | Arctiinae | *Arctium lappa* | MH375874.1 | 152,767 | 37.7 | - |
| Cardueae | Carlininae | *Atractylodes chinensis* | MG874805.1 | 153,258 | 37.7 | - |
| Cardueae | Carlininae | *Atractylodes lancea* | MG874804.1 | 153,201 | 37.7 | - |
| Cardueae | Carlininae | *Atractylodes macrocephala* | MN117071.1 | 153,256 | 37.7 | - |
| Cardueae | Centaureinae | *Carthamus tinctorius* | KM207677.1 | 153,114 | 38.4 | - |
| Cardueae | Centaureinae | *Centaurea diffusa* | KJ690264.1 | 152,559 | 37.8 | - |
| Cardueae | Caduinae | *Cirsium arvense* | KY562583.1 | 152,855 | 37.7 | - |
| Cardueae | Caduinae | *Cirsium eriophorum* | KY562584.1 | 152,557 | 37.7 | - |
| Cardueae | Caduinae | *Cirsium rhinoceros* | MK922360.1 | 152,576 | 37.7 | - |
| Cardueae | Caduinae | *Cirsium vulgare* | KY562585.1 | 152,567 | 37.7 | - |
| Cardueae | Caduinae | *Cynara baetica* | KP842706.1 | 152,548 | 37.7 | - |
| Cardueae | Caduinae | *Cynara cornigera* | KP842707.1 | 152,550 | 37.7 | - |
| Cardueae | Caduinae | *Cynara humilis* | KP299292.1 | 152,585 | 37.7 | - |
| Cardueae | Caduinae | *Silybum marianum* | KT267161.1 | 153,202 | 37.7 | - |
| Cichorieae | Lactucinae | *Lactuca sativa* | AP007232.1 | 152,765 | 37.6 | - |
| Cichorieae | Crepidinae | *Taraxacum officinale* | KU361241.1 | 151,324 | 37.7 | - |

-, not applicable.

**Table S2** The best-fit evolutionary models of 11 partitioning subsets calculated by PartitionFinder. # Sites: the number of sites of the partitioning subsets

| **Subset** | **Best Model** | **# Sites** | **Partition names** |
| --- | --- | --- | --- |
| 1 | GTR+G | 6780 | *accD, rpl33, ccsA, psbT, rpl32, ndhF, matK* |
| 2 | GTR+I+G | 19137 | *rps4, psbN, rpoB, rps11, rpl20, atpA, ycf3, petA, atpE, infA, rps2, ycf2, ndhE, ndhK, rpl36, rpl14, psaC, rps18, atpF, rps14* |
| 3 | GTR+G | 13980 | *petN, ndhG, psbH, psaJ, ndhD, psbL, ndhA, psbM, petL, rps8, rps3, rpoC2, rpoC1, ndhH, atpB* |
| 4 | GTR+I+G | 8016 | *atpH, psbE, psbA, ndhJ, psbJ, psbC, psaA, psaB* |
| 5 | GTR+G | 4767 | *psbB, petG, psaI, psbK, ycf4, atpI, ndhC, psbD, psbF* |
| 6 | GTR | 690 | *cemA* |
| 7 | GTR+G | 3144 | *clpP, rps19, ndhI, rps15, rpoA, rpl22* |
| 8 | GTR | 3432 | *psbZ, psbI, ndhB, rpl2, rps7, rpl23* |
| 9 | GTR+I+G | 2136 | *petB, rbcL* |
| 10 | GTR+I+G | 1608 | *petD, rps12, rpl16, rps16* |
| 11 | GTR+G | 5856 | *ycf1* |

**Table S3** Phylogenetic informativeness profiles for 79 plastid protein-coding genes. Included are number of sites for which substitution rates were calculated (#Rates), net phylogenetic informativeness at epochs: 0-0.015 (Net PI) and per-site phylogenetic informativeness (Per-site PI). Ten genes with the most net informativeness are colored in red

| **Loci** | **#Rates** | **Mean rate** | **SD** | **Net PI** | **Per-site PI** |
| --- | --- | --- | --- | --- | --- |
| *ycf1* | 5592 | 5.644 | 39.677 | 415.361 | 0.0743 |
| *rpoC2* | 4203 | 0.97 | 3.998 | 61.588 | 0.0147 |
| *ndhF* | 6930 | 1.796 | 7.53 | 58.928 | 0.0262 |
| *ycf2* | 2247 | 0.539 | 3.519 | 54.611 | 0.0079 |
| *rpoB* | 3231 | 0.781 | 3.851 | 38.367 | 0.0119 |
| *matK* | 1518 | 1.507 | 5.006 | 34.41 | 0.0227 |
| *accD* | 1461 | 1.485 | 4.858 | 32.678 | 0.0224 |
| *rbcL* | 2070 | 1.476 | 8.955 | 29.283 | 0.0202 |
| *rpoC1* | 687 | 0.894 | 4.064 | 27.278 | 0.0132 |
| *ccsA* | 1023 | 1.705 | 7.078 | 24.018 | 0.0249 |
| *atpB* | 1527 | 1.049 | 5.071 | 23.451 | 0.0157 |
| *ndhD* | 1524 | 0.931 | 4.172 | 21.365 | 0.0140 |
| *rpoA* | 1494 | 5.723 | 140.897 | 21.352 | 0.0209 |
| *ndhA* | 1179 | 1.129 | 5.282 | 17.883 | 0.0164 |
| *ndhH* | 966 | 1.012 | 4.061 | 17.778 | 0.0151 |
| *cemA* | 1089 | 1.744 | 4.701 | 16.922 | 0.0246 |
| *psbB* | 2250 | 0.736 | 3.286 | 16.546 | 0.0109 |
| *atpA* | 2202 | 0.673 | 3.863 | 15.615 | 0.0102 |
| *psaA* | 1524 | 0.46 | 2.826 | 15.577 | 0.0069 |
| *psaB* | 1452 | 0.433 | 2.728 | 14.29 | 0.0065 |
| *petB* | 1419 | 1.425 | 9.923 | 11.786 | 0.0172 |
| *clpP* | 498 | 1.201 | 4.597 | 11.093 | 0.0188 |
| *ndhI* | 960 | 1.375 | 5.358 | 10.054 | 0.0202 |
| *psbD* | 654 | 0.607 | 3.228 | 9.861 | 0.0093 |
| *rpl22* | 1059 | 1.34 | 5.26 | 9.675 | 0.0205 |
| *petA* | 528 | 0.669 | 3.193 | 9.58 | 0.0100 |
| *rps3* | 591 | 1.003 | 4.875 | 9.504 | 0.0145 |
| *psbC* | 471 | 0.452 | 2.747 | 9.435 | 0.0066 |
| *petD* | 603 | 1.588 | 11.871 | 9.296 | 0.0174 |
| *rpl16* | 708 | 1.744 | 9.981 | 9.193 | 0.0225 |
| *ndhK* | 552 | 0.663 | 5.036 | 7.968 | 0.0094 |
| *rps16* | 402 | 30.131 | 461.545 | 7.934 | 0.0301 |
| *rps12* | 684 | 1.529 | 10.087 | 7.249 | 0.0196 |
| *ndhG* | 276 | 0.887 | 3.67 | 6.859 | 0.0130 |
| *rps2* | 741 | 0.619 | 3.115 | 6.527 | 0.0092 |
| *atpI* | 846 | 0.563 | 3.082 | 6.37 | 0.0086 |
| *rps4* | 1059 | 0.678 | 3.015 | 5.923 | 0.0098 |
| *rps8* | 204 | 0.937 | 3.755 | 5.61 | 0.0140 |
| *rps15* | 408 | 1.285 | 4.255 | 5.226 | 0.0189 |
| *rps19* | 276 | 1.18 | 4.577 | 5.052 | 0.0183 |
| *ycf4* | 576 | 0.643 | 2.84 | 5.051 | 0.0092 |
| *psbA* | 264 | 0.305 | 2.285 | 4.925 | 0.0047 |
| *rpl33* | 408 | 1.555 | 5.614 | 4.821 | 0.0236 |
| *ycf3* | 1530 | 0.644 | 4.754 | 4.793 | 0.0095 |
| *atpF* | 162 | 12.165 | 282.02 | 4.467 | 0.0078 |
| *rpl20* | 366 | 0.655 | 3.445 | 3.881 | 0.0103 |
| *rpl32* | 534 | 1.522 | 5.599 | 3.698 | 0.0228 |
| *ndhC* | 378 | 0.648 | 3.477 | 3.65 | 0.0101 |
| *rps11* | 399 | 0.583 | 2.909 | 3.48 | 0.0085 |
| *rpl2* | 360 | 0.254 | 2.407 | 3.379 | 0.0041 |
| *psbH* | 474 | 0.87 | 4.112 | 3.248 | 0.0137 |
| *atpE* | 504 | 0.53 | 2.911 | 3.206 | 0.0080 |
| *ndhE* | 237 | 0.605 | 3.739 | 3.04 | 0.0100 |
| *psbT* | 231 | 1.803 | 9.414 | 2.665 | 0.0247 |
| *ndhJ* | 303 | 0.373 | 2.414 | 2.644 | 0.0056 |
| *ndhB* | 93 | 0.12 | 1.246 | 2.599 | 0.0017 |
| *rpl14* | 465 | 0.465 | 2.431 | 2.422 | 0.0066 |
| *infA* | 300 | 0.572 | 3.009 | 1.973 | 0.0085 |
| *psaC* | 243 | 0.5 | 2.875 | 1.84 | 0.0076 |
| *psaJ* | 825 | 0.839 | 4.19 | 1.788 | 0.0135 |
| *rps14* | 93 | 0.396 | 2.494 | 1.788 | 0.0060 |
| *psbM* | 105 | 1.025 | 4.199 | 1.629 | 0.0155 |
| *rps7* | 129 | 0.226 | 1.714 | 1.5 | 0.0032 |
| *petL* | 303 | 1.127 | 3.699 | 1.491 | 0.0160 |
| *rps18* | 108 | 0.342 | 2.083 | 1.465 | 0.0048 |
| *psbK* | 369 | 0.503 | 3.41 | 1.38 | 0.0078 |
| *psbE* | 177 | 0.322 | 2.368 | 1.243 | 0.0050 |
| *psbL* | 108 | 0.59 | 3.795 | 1.15 | 0.0101 |
| *psbN* | 132 | 0.599 | 2.728 | 1.09 | 0.0085 |
| *psbF* | 111 | 0.585 | 3.4 | 1.08 | 0.0092 |
| *petN* | 111 | 0.813 | 3.113 | 1.053 | 0.0113 |
| *atpH* | 279 | 0.275 | 2.298 | 1.051 | 0.0043 |
| *psbJ* | 249 | 0.445 | 2.932 | 0.86 | 0.0072 |
| *rpl23* | 243 | 0.192 | 1.599 | 0.772 | 0.0028 |
| *petG* | 117 | 0.483 | 2.512 | 0.771 | 0.0069 |
| *rpl36* | 189 | 0.474 | 2.465 | 0.749 | 0.0068 |
| *psaI* | 120 | 0.478 | 2.453 | 0.728 | 0.0067 |
| *psbZ* | 114 | 0.139 | 1.348 | 0.374 | 0.0020 |
| *psbI* | 108 | 0.116 | 1.205 | 0.174 | 0.0016 |
